# Facile Green Synthesis of BiOBr Nanostructures with Superior Visible-Light-Driven Photocatalytic Activity

**DOI:** 10.3390/ma11081273

**Published:** 2018-07-24

**Authors:** Seema Garg, Mohit Yadav, Amrish Chandra, Sameer Sapra, Soniya Gahlawat, Pravin P. Ingole, Milica Todea, Eniko Bardos, Zsolt Pap, Klara Hernadi

**Affiliations:** 1 Department of Chemistry, Amity Institute of Applied Sciences, Amity University, Sector-125, Noida 201313, Uttar Pradesh, India; yadavmohit27@gmail.com; 2 Amity Institute of Nanotechnology, Amity University, Sector-125, Noida 201313, Uttar Pradesh, India; 3 Amity Institute of Pharmacy, Amity University, Sector-125, Noida 201313, Uttar Pradesh, India; chandra.amrish@gmail.com; 4 Department of Chemistry, Indian Institute of Technology, Delhi, Hauz Khas, New Delhi 110016, India; sapra@chemistry.iitd.ac.in (S.S.); soniyagh.iitd@gmail.com (S.G.); ppingole@chemistry.iitd.ac.in (P.P.I.); 5 Nanostructured Materials and Bio-Nano-Interfaces Center, Interdisciplinary Research Institute on Bio-Nano-Sciences, Babeș-Bolyai University, Cluj-Napoca 400271, Romania; militodea@yahoo.com (M.T.); k.pap.zsolt@gmail.com (Z.P.); 6 Department of Molecular Sciences, Faculty of Medicine, Iuliu Haţieganu University of Medicine and Pharmacy, Cluj-Napoca 400012, Romania; 7 Department of Applied and Environmental Chemistry, University of Szeged, Rerrich tér 1, H-6720 Szeged, Hungary; bardosenci@gmail.com (E.B.); hernadi@chem.u-szeged.hu (K.H.); 8 Institute of Environmental Science and Technology, University of Szeged, Tisza Lajos krt. 103, H-6720 Szeged, Hungary

**Keywords:** bismuth oxybromide, phenol, methyl orange, photocatalysis, hydrolysis

## Abstract

Novel green bismuth oxybromide (BiOBr-G) nanoflowers were successfully synthesized via facile hydrolysis route using an *Azadirachta indica* (Neem plant) leaf extract and concurrently, without the leaf extract (BiOBr-C). The *Azadirachta indica* leaf extract was employed as a sensitizer and stabilizer for BiOBr-G, which significantly expanded the optical window and boosted the formation of photogenerated charge carriers and transfer over the BiOBr-G surface. The photocatalytic performance of both samples was investigated for the degradation of methyl orange (MO) and phenol (Ph) under the irradiation of visible light. The leaf extract mediated BiOBr-G photocatalyst displayed significantly higher photocatalytic activity when compared to BiOBr-C for the degradation of both pollutants. The degradation rate of MO and Ph by BiOBr-G was found to be nearly 23% and 16% more when compared to BiOBr-C under visible light irradiation, respectively. The substantial increase in the photocatalytic performance of BiOBr-G was ascribed to the multiple synergistic effects between the efficient solar energy harvesting, narrower band gap, high specific surface area, porosity, and effective charge separation. Furthermore, BiOBr-G displayed high stability for five cycles of photocatalytic activity, which endows its practical application as a green photocatalyst in the long run.

## 1. Introduction

Over the years, visible light-driven semiconductors have gained a lot of interest because of their efficient photocatalytic performance and higher stability for the photodegradation of organic pollutants. In the recent past, titanium dioxide was the best-known photocatalyst for the degradation of organic pollutants. However, due to its wide band gap (3.2 eV), its photocatalytic performance is confined under natural sunlight [1,2,3,4]. Until now, various approaches have been employed to enhance the photocatalytic performance of TiO_2_ under visible light such as doping with the transition metal or non-metals [5,6,7], coupling with narrow band gap semiconductors [8,9], dye-sensitization [10,11], etc. However, in certain cases, the functionalization of TiO_2_ has resulted in self photodegradation, instability under visible light, and residual toxicity [12].

To make optimum use of the visible light, bismuth oxyhalides [13,14] BiOX (X = Cl, Br, I), a new class of visible light active semiconductors have been extensively investigated for environmental remediation and photocatalytic energy conversion. They are non-toxic, chemically stable and possess a unique optical property. Among the BiOX, BiOBr is profoundly utilized due to its high photocatalytic activity, suitable band gap, and stability under visible light illumination. The intrinsic crystalline layer and lamellar structure of BiOBr makes it a suitable photocatalyst [15,16,17]. Regardless of these superior advantages, the application of bismuth oxybromide is practically limited because of the high recombination of the photogenerated e^−^-h^+^ pairs and inadequate light absorption efficiency [18]. To overcome these limitations, various approaches have been adopted to modulate and tailor BiOBr such as the exposure of different crystal faces [19,20], morphological control [21,22], and heterogeneous hybridization [23,24]. However, most of these techniques are not eco-friendly, and some of the methods involve the use of various surfactants and solvents such as CTAB [25], ethanol [26], ethylene glycol [27], and EDTA [28], etc., which could result in residual toxicity and secondary pollution.

Therefore, to eliminate the use of harmful chemicals, a massive increase in the usage of plant extracts has been seen in the synthesis process of various nanostructures. The green method serves as an economical and eco-friendly approach, and eliminates the consumption of expensive and toxic chemicals. Nowadays, different parts of plant extracts have been utilized in the synthesis process such as *Solanum xanthocarpum* Berry [29], tea leaf [30], *Ocimum sanctum* [31], *Aloe vera* [32], *Memecylon edule* [33], *Terminalia chebula* [34], *Arnebia nobilis* [35], etc. One of the most common and important plants, i.e., *Azadirachta indica* (Neem) has several phytochemicals present in the leaf extract such as flavones, ketones, terpenoids, organic acids, aldehydes, amino acids, etc., which mediates the reduction and stabilization of the metal ions into their corresponding nanostructures [36,37]. Benefitting from these properties, various nanostructures using *Azadirachta indica* leaf extract have been synthesized so far such as Ag nanoparticles [38], Fe nanoparticles [39], ZnO nanoparticles [40], titanium nanoparticles [41], etc.

To the best of our knowledge, the synthesis of BiOBr using an *Azadirachta indica* leaf extract or any other leaf extract has not been reported yet. Hence, in our proposed work, we synthesized BiOBr-G using aqueous leaf extract for the first time by the hydrolysis route with minor modifications [42]. The method involves the use of *Azadirachta indica* leaf extract acting as a natural stabilizer and template to reduce the size of nanoplates, enhance the surface area, and inhibition of e^−^-h^+^ pairs recombination under visible light irradiation to enhance the photocatalytic performance of BiOBr-G.

## 2. Materials and Methods

### 2.1. Chemicals

Analytical grade chemicals like potassium bromide, phenol, methyl orange and bismuth nitrate [Bi(NO_3_)_3_·5H_2_O] were procured from Merck India.

### 2.2. Synthesis of Plant Extract

Fresh *Azadirachta indica* leaves were collected from the Amity University campus, Noida, India. The leaves were cleaned thoroughly with running tap water followed by double distilled water to remove debris and other impurities and dried at room temperature. A total of 10 g of chopped leaves was added to 100 mL double distilled water in a 250 mL beaker and allowed to boil on a hotplate for 30 min. The extract was cooled down, filtered ,and utilized for the synthesis process.

### 2.3. Synthesis of BiOBr-G and BiOBr-C

In a typical experimental procedure, 2.425 g of bismuth nitrate was dissolved in 100 mL ouble distilled water containing 10 mL glacial acetic acid, and the resulting solution was added to 8 mL plant extract (Supporting Information, Section S1) under magnetic stirring. Then, the obtained solution was added dropwise to a 100 mL KBr solution (0.595 g of KBr dissolved in 100 mL water) under vigorous stirring. At room temperature, it was stirred for 1 h and kept in an oven for 1 h at 60 °C. The resulting suspension was centrifuged to separate the product and then washed three times with ethanol and double distilled water to remove the impurities. Finally, it was dried in an oven at 60 °C. BiOBr-C was synthesized through the same procedure; however, the use of plant extract was avoided. (Supporting Information, Figure S2).

### 2.4. Characterization

The X-ray diffraction (XRD) patterns using a Bruker D2-Phaser Diffractometer (Coventry, UK) with a Cu Kα radiation source (λ = 1.5418 Å) were recorded. The surface morphology and elemental analysis of BiOBr-G and BiOBr-C were examined by Zeiss-Sigma VP FESEM, Ostalbkreis, Germay (field emission scanning electron microscope), FEI Technai G2 X-Twin TEM, Malaga, Spain (transmission electron microscope), 200 kV and EDS (energy dispersive X-ray spectroscopy), Bruker, Coventry, UK. Surface area was estimated by the BET method for which nitrogen adsorption–desorption studies were carried out at 77 K using a Quanta Chrome NOVA 1000, Graz, Austria. XPS (X-ray photoelectron spectroscopy) data were obtained on a PHOIBOS (150 MCD) device (Berlin, Germany) with 1486.69 eV, Al K_α_ monochromatic radiation at 20 mA and 14 kV, and the pressure <10^−9^ mbar. The functional groups on the as-prepared samples were determined using FTIR (Agilent, Cary 630, NC, USA). The optical characteristics were analyzed by both UV-vis diffuse reflectance spectra (Shimadzu UV-1800, Columbia, SC, USA) and photoluminescence spectra (Shimadzu RF-5301, Columbia, SC, USA).

### 2.5. Photocatalytic Experiment

The photocatalytic experiment was conducted in a photoreactor equipped with four compact fluorescent lamps 28 W each to provide visible light illumination to the photocatalyst. A 50 g L^−1^ NaNO_2_ solution was used as a UV cut off filter [43]. Initially, 50 mg of the photocatalyst was added to a 100 mL aqueous solution of phenol and methyl orange with an initial concentration of 50 mg L^−1^ and 20 mg L^−1^, respectively. For the control experiment, 10 mL leaf extract was added to 90 mL aqueous solution of 20 mg L^−1^ MO. Before illumination, the slurry was agitated in the dark for 60 min to ensure adequate adsorption–desorption equilibrium (Supporting Information, Section S3, Figure S3). The slurry was then subjected to visible light illumination under constant stirring. At certain time periods, a 3 mL sample was taken from the reactor and separated via centrifugation. The absorbance of the supernatant was measured by UV-vis spectrophotometer at 465 nm for MO and 270 nm for Ph. The concentration of the photocatalyst varied from 50–125 mg for MO and 50–150 mg for Ph, respectively. The intermediates generated by photolysis of phenol were detected by high-performance liquid chromatography (HPLC, Shimadzu, Kyoto, Japan). Reversed phase column (Enable C18G, 25 × 0.46 cm with an internal diameter of 5 µm) and a mobile phase consisting of a mixture of methanol (60% *v*/*v*) and water (40% *v*/*v*) was used and a 1.0 mL/min flow rate was set. The intermediates were identified by a 6520 Accurate-Mass Q-TOF LC-MS (liquid chromatography-mass spectroscopy), Agilent Technologies instrument. The range was *m*/*z* 50–500, and +ve ions were monitored.

### 2.6. Radical Trapping Experiment

Individual scavengers (1 mM isoprapanol, 1 mM benzoquinone, and 1 mM sodium oxalate) were added to determine the active species responsible for the photocatalytic activity i.e., hydroxyl radicals (OH), superoxide radicals (O_2_^−^), and holes (h**^+^**), respectively. The same methodology of the photocatalytic experiment was followed as above-mentioned.

### 2.7. Electrochemical Measurements

The electrochemical studies were carried out on a CHI 608E potentiostat (CHI Instruments, Austin, TX, USA) equipped with 150 W Xe arc lamp (Optosolar GmbH, Merdingen, Germany) using the standard three electrode system, a working electrode (catalyst ink mounted on ITO substrate), a saturated Hg/HgO electrode (reference electrode), and a platinum wire (counter electrode). To prepare the working electrode, the drop casting method was adopted: briefly, 2 mg of the material was ultrasonically suspended in a mixture of 100 µL ethanol and 10 µL nafion, of which 10 µL was then drop-casted on a piece of ITO coated glass substrate with a fixed area of 1 cm^2^. A 0.1 M KOH (pH 12.8) was used as the electrolyte for the photoelectrochemical (PEC) studies. Transient photocurrent response was recorded at 0.0 V vs. Hg/HgO reference electrode. Electrochemical impedance spectroscopy (EIS) measurements were also carried out at 0.0 V versus Hg/HgO within frequency range of 1 MHz to 10 mHz.

## 3. Results and Discussion

### 3.1. XRD Analysis

**Figure 1:**
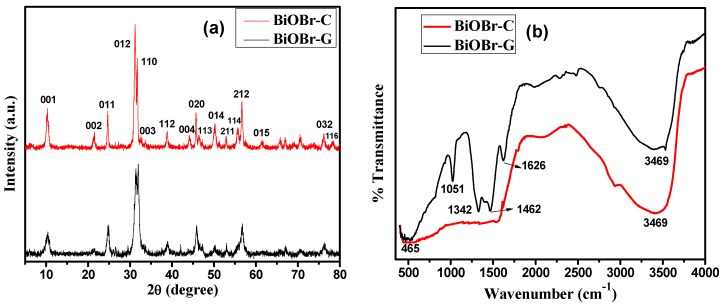
(**a**) XRD patterns; (**b**) FTIR spectra of BiOBr-C and BiOBr-G.

Figure 1a shows the X-ray diffraction patterns of the as-prepared BiOBr-G and BiOBr-C. All the identified peaks corresponding to (001), (002), (011), (012), (110), (003), (112), (004), (020), (113), (014), (211), (114), (212), (015), (032) ,and (116) planes are according to the JCPDS Card No. 73-2061 [44] and can be indexed to the tetragonal phase of BiOBr. Interestingly, with the incorporation of the leaf extract in BiOBr-G, the overall intensity of the XRD peaks decreased, suggesting the slight degradation of crystallinity. The most probable reason behind the decrease in crystallinity could be the generation of crystal defects in the BiOBr-G lattice, which resulted in the charge imbalance and changed stoichiometry of the sample [45].

In addition, no additional peaks and chemical changes in the crystal structure of BiOBr-G were observed, indicating a high purity of the sample. Furthermore, in BiOBr-G, the full width at half maximum (FWHM) of the XRD peaks seemed to be widened, indicating a smaller crystallite size than BiOBr-C. The crystallite sizes of BiOBr-G and BiOBr-C were calculated using Debye Scherer’s Equation (Supporting Information, Section S3) and were found to be 6.9 nm and 17.2 nm, respectively.

### 3.2. FTIR Analysis

In order to investigate the functional groups present in BiOBr-C and BiOBr-G, FTIR spectra were carried out as shown in Figure 1b. The absorption peak originating at 3469 cm^−1^ was assigned to the O–H stretching vibration mode of water. The peak at 465 cm^−1^ in the spectra of BiOBr-C and BiOBr-G was attributed to the Bi–O chemical stretching vibration mode in the crystal tetragonal phase of BiOBr, respectively [46]. Some additional peaks were also observed in the spectra of BiOBr-G, the peaks originated at 1626 cm^−1^ and 1051 cm^−1^ were ascribed to the amide C=O stretching and C–O–C linkages or C–O bonds [40]. The peaks at 1342 cm^−1^ and 1462 cm^−1^ accounted for the C–OH and O=C–H vibrations, respectively. The additional peaks were mainly attributed to the flavonoids and other phytochemicals present markedly in the *Azadirachta indica* leaf extract [36,41].

### 3.3. XPS Analysis

**Figure 2:**
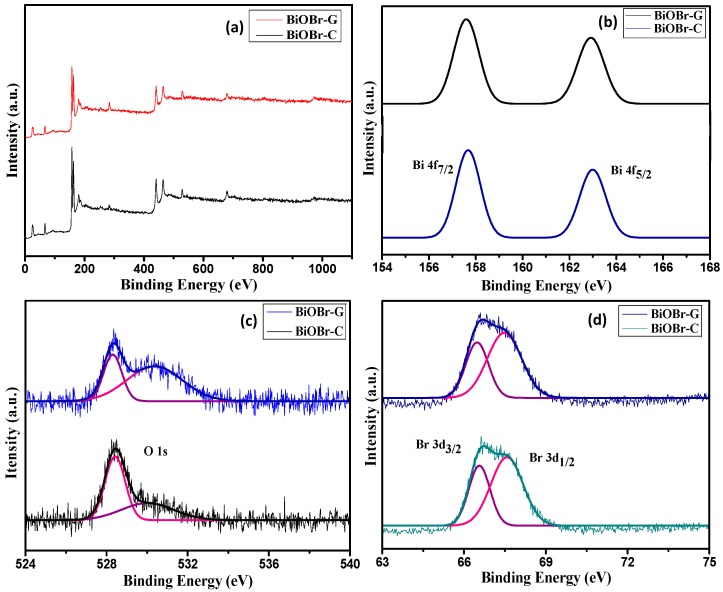
XPS spectra (**a**) Survey of the samples, (**b**) Bi 4f, (**c**) O 1s, and (**d**) Br 3d, of BiOBr-C and BiOBr-G.

The chemical composition and surface chemical states of the as-prepared BiOBr-G and BiOBr-C were evaluated by XPS as shown in Figure 2. As shown in Figure 2a, Bi, O, and Br peaks were displayed in the XPS spectra of both materials, which was in accordance with the chemical constituent of the materials. Figure 2b shows the Bi 4f high-resolution spectra of both samples; 157.9 eV and 163.3 eV peaks were assigned to Bi 4f_7/2_ and Bi4f_5/2_, which indicated the existence of Bi^3+^ in both samples [47]. The high-resolution O 1s XPS spectra of the BiOBr-G and BiOBr-C are displayed in Figure 2c. For BiOBr-C, the O 1s profile fitted into two symmetrical peaks located at 528.3 eV and 530.1 eV, indicating two different oxygen (O) species in the sample. The O 1s peak at 528.3 eV was ascribed to the O atoms (Bi–O crystal lattice). The peak at 530.1 eV was ascribed to the bismuth–oxygen bonds in the [Bi_2_O_2_] slabs of BiOBr. In the high-resolution O 1s spectrum of BiOBr-G, the peak at 530.3 eV was shifted marginally, demonstrating a minor change in the oxygen environment [15,48] due to the incorporation of *Azadirachta indica* in the BiOBr-G. The high-resolution XPS Br 3d spectra (Figure 2d) shows two distinct peaks at a binding energy of 66.6 eV and 67.5 eV, and were attributed to Br 3d_3/2_ and Br 3d_1/2_, respectively [49]. The results were consistent with the XRD analysis.

### 3.4. Morphology Study

**Figure 3:**
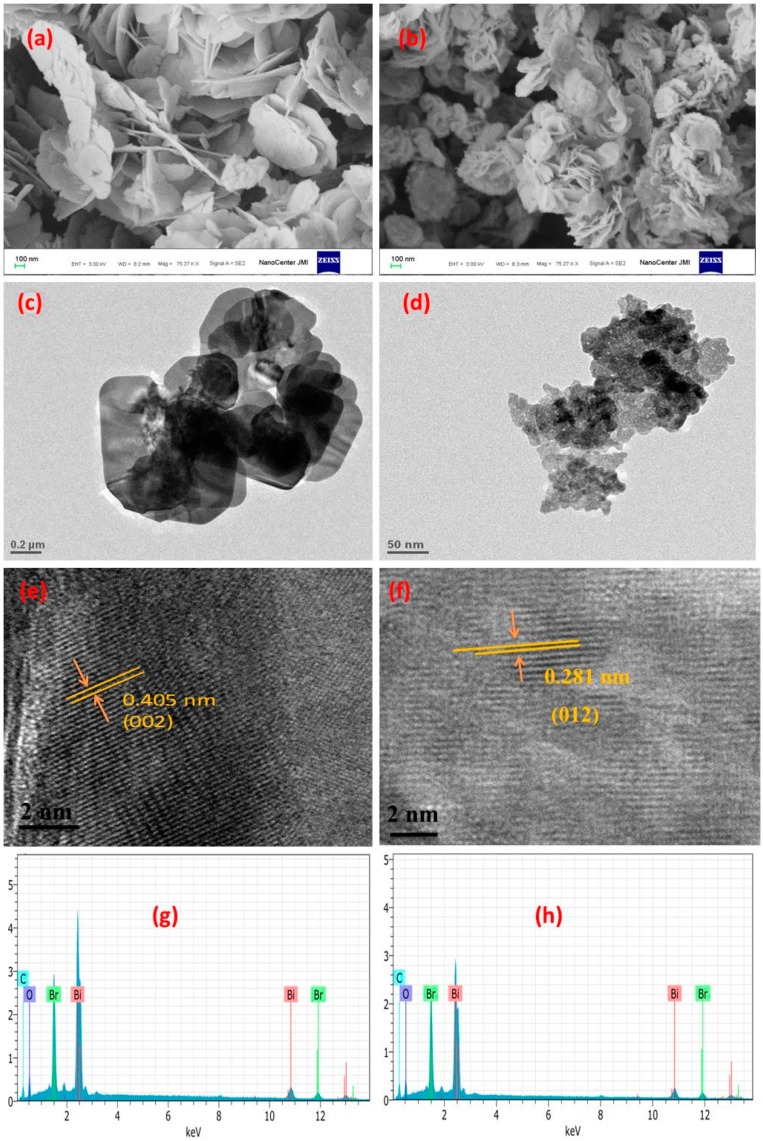
(**a**,**b**) FESEM images, (**c**,**d**) TEM images, (**e**,**f**) HR-TEM images, and (**g**,**h**) EDS images of BiOBr-C (**left**) and BiOBr-G (**right**).

The morphology studies of BiOBr-C and BiOBr-G were examined by FESEM and TEM. It can be evidently seen in Figure 3a,b that there was a larger difference in the size of the BiOBr-C and BiOBr-G nanoplates. BiOBr-G had smaller nanoplates when compared to BiOBr-C. This suggests that *Azadirachta indica* leaves successfully reduced the size of the plates of BiOBr-G and could enhance the specific surface area.

To study the detailed structure of BiOBr-C and BiOBr-G, TEM investigations were carried out as shown in Figure 3c,d, respectively. The TEM images further confirmed the smaller size of BiOBr-G nanoplates with the addition of internal cavities in comparison to the BiOBr-C nanoplates (Supporting Information, Section S5, Figure S5). To obtain a deeper view, the as-prepared samples were further characterized with HR-TEM as shown in Figure 3e,f. Clearly, a highly crystalline pattern was observed with clear lattice fringes in both samples. The continuous lattice fringes with an interplanar lattice spacing of 0.405 nm and 0.281 nm matched well with the (002) and (012) atomic planes of BiOBr. The existence of the smaller nanoplates and internal cavities of BiOBr-G nanostructures could result in multiple reflections of the irradiated light and subsequently increase the photocatalytic activity [50].

In addition, the chemical composition of the BiOBr-C and BiOBr-G samples were also analyzed by EDS as shown in Figure 3g,h, respectively. The results indicate that both BiOBr-C and BiOBr-G contained elements of Bi, Br, and O.

### 3.5. Specific Surface Area Analysis

**Figure 4:**
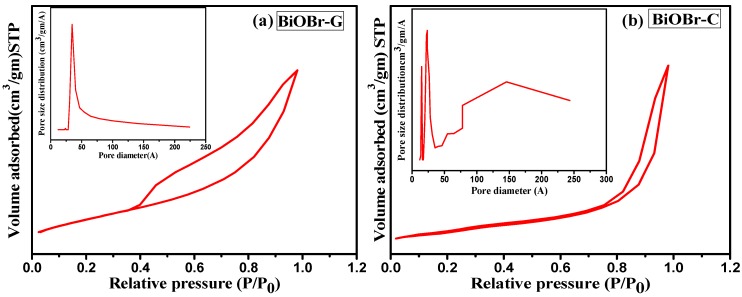
Nitrogen adsorption–desorption isotherms and pore size distribution for (**a**) BiOBr-C and (**b**) BiOBr-G.

The specific surface area and pore size distribution of the as-prepared BiOBr-G and BiOBr-C samples were calculated by the nitrogen adsorption–desorption study as shown in Figure 4a,b. It can be seen that both the photocatalysts exhibited the type IV isotherm with a distinct H3 hysteresis loop at high relative pressure indicating a mesoporous structure according to the IUPAC classification [51,52]. The BET specific surface area and pore volume for BiOBr-C were found to be 13.938 m^2^/g and 0.039 m^3^/g, and 79.592 m^2^/g and 0.116 m^3^/g for BiOBr-G, respectively. The average Barett–Joyner–Halenda (BJH) pore diameter of the BiOBr-G and BiOBr-C calculated from desorption isotherm study was 2.358 nm and 3.442 nm, respectively. In the case of BiOBr-G, a uniform pore size distribution was observed, in contrast, an abrupt and non-uniform pattern was seen for BiOBr-C. Clearly, *Azadirachta indica* leaves play a key role in enhancing the surface area of BiOBr-G, which was up to six times that of BiOBr-C. The uniform distribution of pore size and higher surface area of BiOBr-G could effectively enhance the area of contact and subsequently, facilitate the reactant species to be transferred, resulting in enhanced photocatalytic activity [53].

### 3.6. Optical Absorption Properties

**Figure 5:**
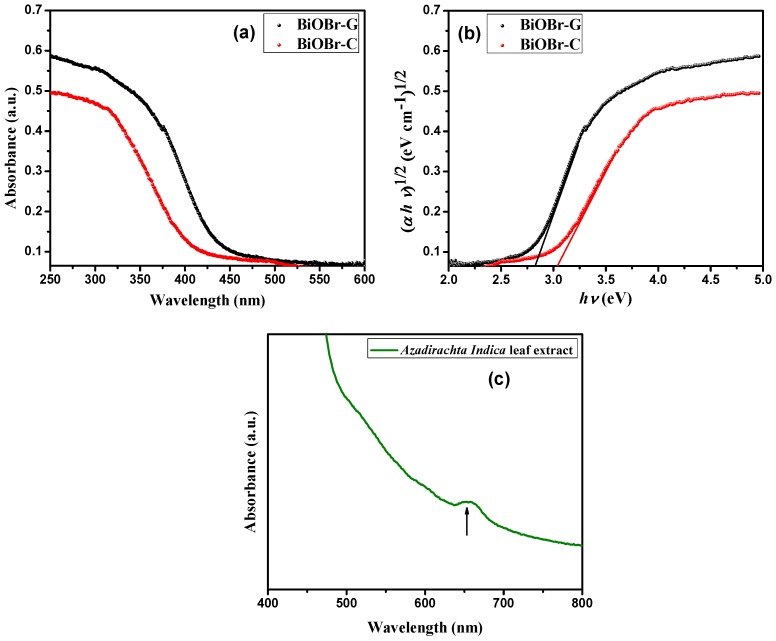
(**a**) UV-vis diffuse reflection spectra, (**b**) Plot of (αhυ)^1/2^ versus hυ of the BiOBr-C and BiOBr-G, and (**c**) UV-vis absorption spectra of the raw *Azadirachta indica* leaf extract.

The optical absorption property of a visible light-driven photocatalyst is a major factor in determining its photocatalytic activity. Figure 5a displays the UV-vis diffuse reflection spectra (DRS) of BiOBr-C and BiOBr-G, respectively. Clearly, the absorption edges of both photocatalysts were positioned in the visible light range (<<410 nm). BiOBr-G displayed greater absorption and a red shift when compared to BiOBr-C; this shift could be attributed to the action of the *Azadirachta indica* leaf extract. The optical absorption region of semiconductors can be evaluated by the energy of band gap (E_g_) and analyzed by the following equation:

αhυ = A(hυ − E_g_)^n/2^ (1)

where E_g_, υ, α, and A are the band gap energy, frequency of light, absorption coefficient, and a constant, respectively, for direct transition (*n* = 1) and for indirect transition (*n* = 4). The n value was 4 for BiOBr [54]. The Tauc plots of BiOBr-C and BiOBr-G are shown in Figure 5b. The E_g_ values of BiOBr-C and BiOBr-G were calculated by the extrapolation of these lines to the photon energy axis, and were found to be 3.04 eV and 2.83 eV, respectively. The lower band gap in BiOBr-G facilitated the electronic transitions and visible light response towards the organic pollutants. Furthermore, the UV-visible absorption spectra of the raw *Azadirachta indica* leaf extract was evaluated in the wavelength range of 400–800 nm to confirm the presence of phytochemicals in the leaf extract. As shown in Figure 5c, the maximum absorbance of the leaf extract was observed in the range of 600–700 nm, which indicated the existence of complex organic molecules carrying different charge centers. Therefore, the phytochemicals in the leaf extract tend to capture more visible light, which can boost the formation of large number of photogenerated electron–hole pairs for higher photocatalytic activity [40,55,56].

### 3.7. Photocatalytic Performance of BiOBr-C and BiOBr-G

**Figure 6:**
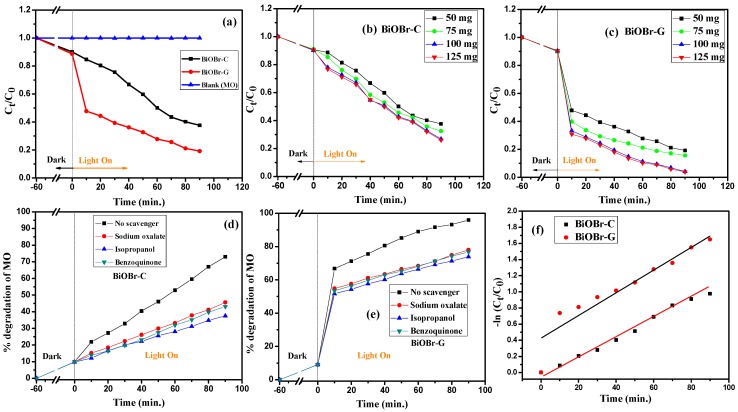
Photodegradation efficiency of MO by BiOBr-C and BiOBr-G (**a**) with time, (**b**) with different concentrations of BiOBr-C, (**c**) with different concentrations of BiOBr-G, (**d**) Effects of scavengers on MO degradation using BiOBr-C, (**e**) Effects of scavengers on MO degradation using BiOBr-G, and (**f**) Kinetic linear simulation curves of MO over the samples.

To examine the photocatalytic activity of the as-prepared samples, 100 mL of MO with an initial concentration of 20 mg L^−1^ was selected as the target dye pollutant. From Figure 6a, it can be observed that no photolysis of MO took place up to 90 min of visible light irradiation in the absence of the photocatalysts, suggesting that MO is chemically stable and has difficulty with self-photolysis. However, in the presence of 50 mg of BiOBr-C and BiOBr-G, the degradation rate of MO was found to be 62.36% and 80.76% at 90 min, respectively.

Furthermore, to evaluate the maximum degradation of MO, the photocatalytic concentration was varied from 50–125 mg. It can be clearly seen in Figure 6b,c that with an increase in photocatalytic concentration, the degradation of MO was further increased up to 73.45% and 95.91% for BiOBr-C and BiOBr-G, respectively. Moreover, the photocatalytic efficiency of BiOBr-G was found to be nearly 23% more than that of BiOBr-C.

A batch of experiments was then carried out to examine the active species responsible for MO degradation under light irradiation (>400 nm) by using scavengers (sodium oxalate for h^+^, isopropanol for ·OH, and benzoquinone for **^.^**O_2_^−^). It was observed that the photocatalytic activity was suppressed in all cases as shown in Figure 6d,e, indicating that all three scavengers played a key role in the photo-oxidation process. Hence, the photocatalytic degradation of MO was expected to occur via oxidation by all the active species^-^, which were produced during the following photo-oxidation process:

BiOBr + hυ → BiOBr + h**^+^** + e^−^ (2)

O_2_ + e^−^ → ·O_2_^−^ (3)

2H_2_O + h**^+^** → 2·OH + 2H^+^ (4)

MO + h**^+^**, **^.^**O_2_^−^ or ·OH → degraded product (5)

The photodegradation process of MO was fit with a pseudo-first order kinetics model as shown in Figure 6f.

−ln (*C_t_*/*C*_0_) = *kt* (6)

where *C_t_* is the MO concentration at time *t*; *C*_0_ is the initial concentration of MO; and *k* is the rate constant. The rate constants for BiOBr-C and BiOBr-G were found to be 0.010 min^−1^ and 0.018 min^−1^, respectively. The higher rate constant means a lower activation energy (E_a_) and higher degradation rate; hence, BiOBr-G demonstrated much higher photodegradation efficiency towards MO when compared to BiOBr-C.

Through the control experiment, it was found that there was no degradation of methyl orange using the leaf extract alone, which suggests that the plant extract alone does not possess any photocatalytic activity (Supporting Information, Section S6).

For the prospect of dye sensitization, a typical colorless pollutant i.e., phenol was also chosen to further assess the photocatalytic performance of BiOBr-C and BiOBr-G (Supporting Information, Section S7).

**Figure 7:**
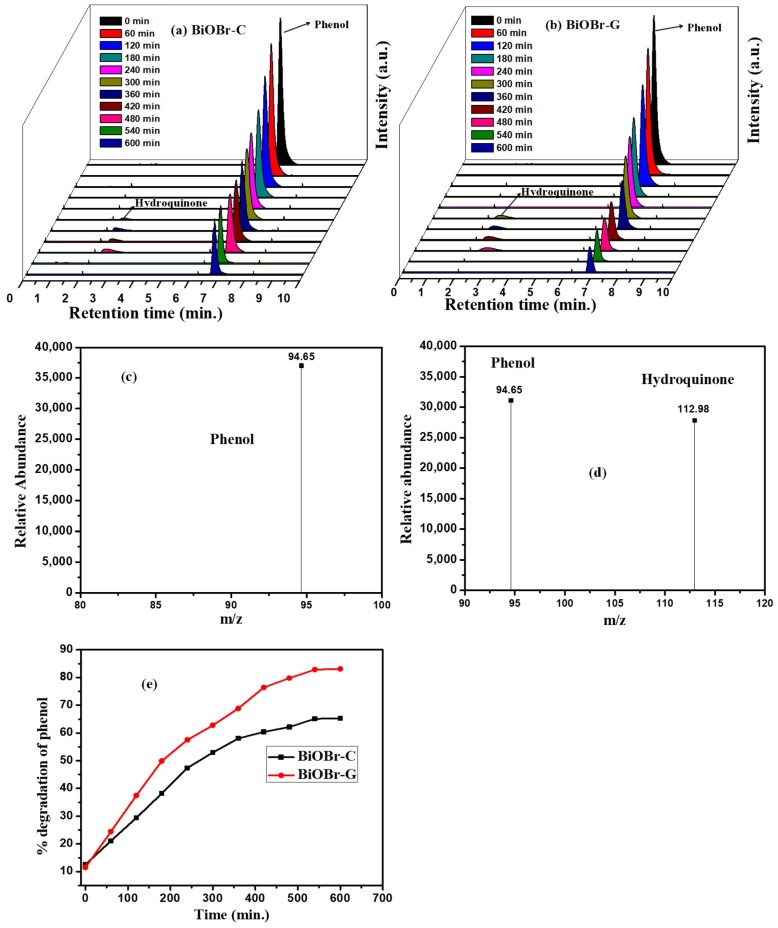
HPLC graphs of phenol degradation by (**a**) BiOBr-C, (**b**) BiOBr-G. Mass spectra of (**c**) phenol, (**d**) phenol and hydroquinone, and (**e**)% degradation of phenol with time by HPLC data.

HPLC was used to detect the reaction intermediates throughout the photocatalytic process for the identification of the degraded products that formed during the photocatalytic degradation of phenol. Figure 7a,b, display the process of the degradation of Ph, where the decreasing peak of Ph was observed at 6.8 min. A growing peak that originated at 2.5 min appeared from the second Ph sample, which was recorded after 300 min of the irradiation under visible light to the sample. The results reveal the existence of hydroquinone as one of the degraded products of Ph by the as-prepared BiOBr-C and BiOBr-G, which was in accordance with the peak occurring at 2.5 min.

To gather detailed information about the reaction intermediate, the samples were analyzed under LC-MS/MS. An obvious peak of Ph was exhibited at *m*/*z* 94.65, while no other peak was observed before irradiation (Figure 7c). However, after 300 min of visible light irradiation, a peak at *m*/*z* 112.98 appeared along with that of phenol as shown in Figure 7d. The results confirmed the existence of phenol and hydroquinone, which were consistent with the literature [57,58]. No peak of hydroquinone was evident after 480 min of visible light irradiation, indicating that the carcinogenic and hematotoxic agent related to malignancy in the occupational environments could also be degraded. Furthermore, Figure 7e shows the percent degradation of the phenol by HPLC results, which also matched well with the previous findings under the UV-vis spectrophotometer.

### 3.8. Reusability and Stability of BiOBr-C and BiOBr-G

**Figure 8:**
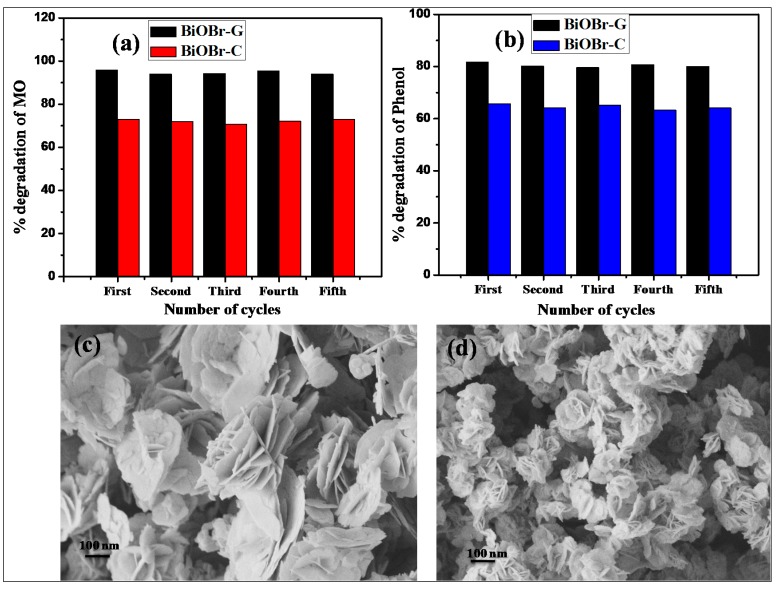
Recyclability of BiOBr-C and BiOBr-G for (**a**) MO and (**b**) Ph; FESEM images after five cycles (**c**) BiOBr-C, and (**d**) BiOBr-G.

In order to examine the stability of BiOBr-C and BiOBr-G, the as-prepared samples after photocatalytic activity with MO and Ph were collected via centrifugation. The materials were washed several times with double distilled water and reused in the photocatalytic reactions five times under the same conditions. As shown in Figure 8a,b, both samples displayed high stability during five reaction cycles. In addition, the FESEM images of both samples after the photocatalytic reaction were examined. As shown in Figure 8c,d, the morphology of the as-prepared BiOBr-C and BiOBr-G remained intact.

### 3.9. Photocatalytic Degradation Mechanism

**Figure 9:**
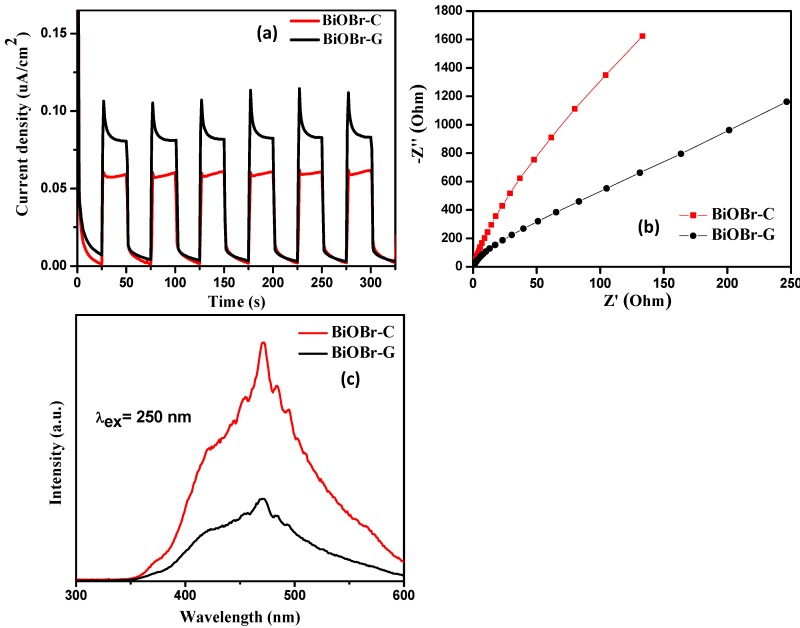
(**a**) Transient photocurrent response, (**b**) EIS Nyquist plots, and (**c**) PL spectra of BiOBr-C and BiOBr-G.

Electrochemical studies were conducted to examine the separation of the photogenerated electron-hole pairs. The transient photocurrent response of the as-prepared BiOBr-C and BiOBr-G were recorded for several ON-OFF cycles of light irradiation. As shown in Figure 9a, upon UV-visible light irradiation, the photocurrent increased sharply and returned rapidly to its initial state when the light irradiation was stopped. The photocurrent response was stable, reproducible, and steady during several sporadic ON-OFF cycles. The BiOBr-G displayed a significantly higher photocurrent response compared to the BiOBr-C, indicating that the recombination of the photogenerated electron-hole pairs was efficiently diminished in BiOBr-G.

Electrochemical impedance spectroscopy (EIS) studies were used to examine the characteristics of the charge transfer and recombination processes in the as-prepared BiOBr-C and BiOBr-G materials. As shown in Figure 9b, the diameter of the arc radius on the EIS Nyquist plot of the BiOBr-C electrode was larger than BiOBr-G electrode under the light irradiation, which revealed a lower recombination of photogenerated e^−^-h^+^ pairs in BiOBr-G compared to BiOBr-C [59,60].

To further examine the migration, recombination, and charge transfer process in the as-prepared BiOBr-C and BiOBr-G, photoluminescence spectra of both the materials were recorded. It can be seen in Figure 9c that BiOBr-G exhibited an emission peak at a much lower intensity than BiOBr-C, which confirmed the successful inhibition of the e^−^-h^+^ pairs recombination in BiOBr-G. The results of the PL analysis were consistent with the electrochemical studies. Therefore, it can be speculated that the synthesis of BiOBr-G using *Azadirachta indica* leaf extract significantly enhanced the photocatalytic activity.

The valence band (VB) and conduction band (CB) of the as-prepared BiOBr-C and BiOBr-G were calculated (Supporting Information, Section S8).

**Figure 10:**
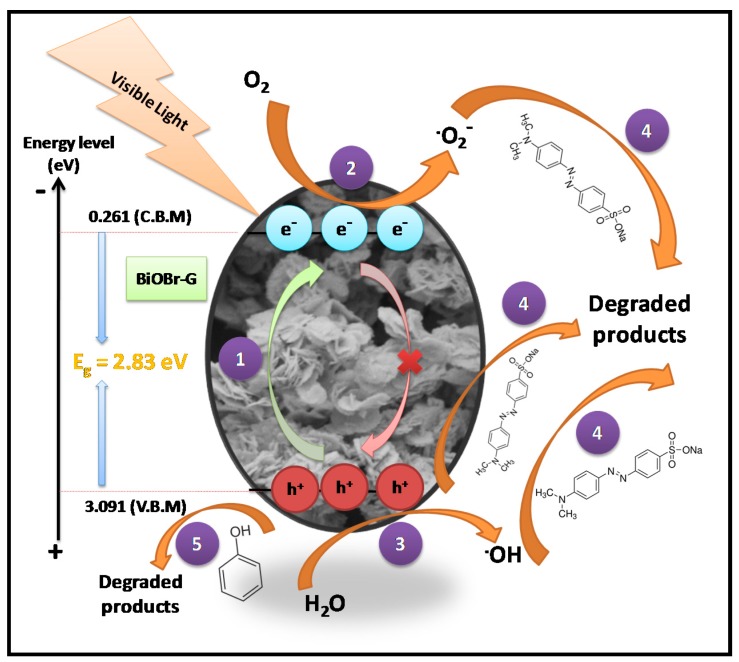
Photocatalytic degradation mechanism of MO and Ph.

Based on the results obtained from the above discussion and scavenger studies, the photocatalytic degradation mechanism of MO and Ph by BiOBr-G was elucidated as shown in Figure 10, respectively. In step ①, the BiOBr-G nanoplates were irradiated by visible light to produce adequate photogenerated carriers, i.e., holes and electrons. In step ②, the excited electrons in the conduction band reacted with the O_2_ molecules, which were adsorbed on the BiOBr-G surface to produce O_2_^−^. The presence of O_2_^−^ inhibited the e^−^-h^+^ pairs recombination, and facilitated the photodegradation of pollutants. In step ③, the remaining holes in the valence band reacted with the H_2_O molecules to produce **^.^**OH radicals. Step ④; which is also the deciding step for the degradation of MO, the major active species, i.e., OH, O_2_^−^ and h**^+^** actively participated in the chemical reaction with the MO molecules to yield the degraded products. In the final step, the photogenerated h**^+^** were the major active species responsible for the photodegradation of Ph to yield the degraded products ⑤ [60,61,62,63].

## 4. Conclusions

The present study demonstrated a novel, eco-friendly, and inexpensive strategy to synthesize BiOBr using *Azadirachta indica* leaf extract. The leaf extract showed a dual nature by acting as an excellent sensitizer and stabilizing template for BiOBr-G. Phytochemicals (present in the leaf extract) were likely to be involved in the enhancement of the optical absorption and formation of the photogenerated electron-hole pairs. In addition, the incorporation of the leaf extract in the BiOBr-G matrix provided a deeper color, which resulted in a narrower band gap of BiOBr-G. As a stabilizing template, the leaf extract effectively controlled the size of the BiOBr-G, which resulted in a higher specific surface area and porosity, thereby exposing more active sites for enhancing the photodegradation of MO and Ph. The electrochemical studies confirmed that the recombination of the photogenerated electron-hole pair was effectively inhibited in BiOBr-G when compared to BiOBr-C. Moreover, the scavenger studies revealed that h^+^ were the main active species responsible for the degradation of phenol, while all three active species (h**^+^**, OH, and O_2_^−^) were found to be responsible for the degradation of methyl orange. The role of plant extracts could provide new understanding into the synthesis of novel green photocatalysts with elevated performance and ease their practical application in environmental problems.

## Supplementary Materials

The following are available online at http://www.mdpi.com/1996-1944/11/8/1273/s1; Figure S1: Photodegradation of MO by BiOBr-G with different concentrations of plant extract, Figure S2: Schematic diagram for synthesis of BiOBr using *Azadirachtaindica* leaf extract, and images of BiOBr-C and BiOBr-G samples, Figure S3: Representation of the dark study at 30 min, 60 min and 90 min for BiOBr-C and BiOBr-G, Section S4: Calculation of Crystallite sizes of BiOBr-C and BiOBr-G, Figure S5: TEM images of BiOBr-C and BiOBr-G, Figure S6: Degradation efficiency of MO with leaf extract, Figure S7: Photodegradation of phenol by BiOBr-C and BiOBr-G, Section S8: Band position calculations of BiOBr-C and BiOBr-G.

## References

[1] Wang T., Yang G., Liu J., Yang B., Ding S., Yan Z., Xiao T. (2014). Orthogonal synthesis, structural characteristics, and enhanced visible-light photocatalysis of mesoporous Fe_2_O_3_/TiO_2_ heterostructured microspheres. Appl. Surf. Sci..

[2] Xue C., Xia J., Wang T., Zhao S., Yang G., Yang B., Dai Y., Yang G. (2014). A facile and efficient solvothermal fabrication of three-dimensionally hierarchical BiOBr microspheres with exceptional photocatalytic activity. Mater. Lett..

[3] Simon G., Gyulavari T., Hernadi K., Molnar M., Pap Z., Vereb G., Schrantz K., Nafradi M., Alapi T. (2018). Photocatalytic ozonation of monuron, over suspended and immobilized TiO_2_-study of transformation, mineralization and economic feasibility. J. Photochem. Photobiol. A.

[4] Vereb G., Ambrus Z., Pap Z., Mogyorosi K., Dombi A., Hernadi K. (2014). Immobilization of crystallized photocatalysts on ceramic paper by titanium (IV) ethoxide and photocatalytic decomposition of Phenol. React. Kinet. Mech. Catal..

[5] He C., Li X., Xiong Y., Zhu X., Liu S. (2005). The enhanced PC and PEC oxidation of formic acid in aqueous solution using a Cu–TiO_2_/ITO film. Chemosphere.

[6] Dai G., Yu J., Liu G. (2011). Synthesis and enhanced visible-light photoelectrocatalytic activity of *p*-*n* junction BiOI/TiO_2_ nanotube arrays. J. Phys. Chem. C.

[7] Liu L., Liu Z., Bai H., Sun D.D. (2012). Concurrent filtration and solar photocatalytic disinfection/degradation using high-performance Ag/TiO_2_ nanofiber membrane. Water Res..

[8] Yang L., Luo S., Li Y., Xiao Y., Kang Q., Cai Q. (2010). High efficient photocatalytic degradation of p-nitrophenol on a unique Cu_2_O/TiO_2_ p-n heterojunction network catalyst. Environ. Sci. Technol..

[9] Su M., He C., Sharma V.K., Asi M.A., Xia D., Li X.-Z., Deng H., Xiong Y. (2012). Mesoporous zinc ferrite: Synthesis, characterization, and photocatalytic activity with H_2_O_2_/visible light. J. Hazard. Mater..

[10] Kim W., Tachikawa T., Majima T., Choi W. (2009). Photocatalysis of dye-sensitized TiO_2_ nanoparticles with thin overcoat of Al_2_O_3_: Enhanced activity for H_2_ production and dechlorination of CCl_4_. J. Phys. Chem. C.

[11] Ushiroda S., Ruzycki N., Lu Y., Spitler M., Parkinson B. (2005). Dye sensitization of the anatase (101) crystal surface by a series of dicarboxylated thiacyanine dyes. J. Am. Chem. Soc..

[12] Li G., Zhang D., Yu J.C., Leung M.K. (2010). An efficient bismuth tungstate visible-light-driven photocatalyst for breaking down nitric oxide. Environ. Sci. Technol..

[13] Xia J., Di J., Li H., Xu H., Li H., Guo S. (2016). Ionic liquid-induced strategy for carbon quantum dots/BiOX (X = Br, Cl) hybrid nanosheets with superior visible light-driven photocatalysis. Appl. Catal. B Environ..

[14] Di J., Xia J., Li H., Guo S., Dai S. (2017). Bismuth oxyhalide layered materials for energy and environmental applications. Nano Energ..

[15] Di J., Xia J., Ji M., Yin S., Li H., Xu H., Zhang Q., Li H. (2015). Controllable synthesis of Bi_4_O_5_Br_2_ ultrathin nanosheets for photocatalytic removal of ciprofloxacin and mechanism insight. J. Mater. Chem. A.

[16] Li J., Yu Y., Zhang L. (2014). Bismuth oxyhalide nanomaterials: Layered structures meet photocatalysis. Nanoscale.

[17] Yu H., Huang H., Xu K., Hao W., Guo Y., Wang S., Shen X., Pan S., Zhang Y. (2017). Liquid-phase exfoliation into monolayered BiOBr nanosheets for photocatalytic oxidation and reduction. ACS Sustain. Chem. Eng..

[18] Di J., Xia J., Yin S., Xu H., Xu L., Xu Y., He M., Li H. (2014). Preparation of sphere-like g-C_3_N_4_/BiOI photocatalysts via a reactable ionic liquid for visible-light-driven photocatalytic degradation of pollutants J. Mater. Chem. A.

[19] Zhang D., Li J., Wang Q., Wu Q. (2013). High {001} facets dominated BiOBr lamellas: Facile hydrolysis preparation and selective visible-light photocatalytic activity. J. Mater. Chem. A.

[20] Zhang H., Yang Y., Zhou Z., Zhao Y., Liu L. (2014). Enhanced photocatalytic properties in BiOBr nanosheets with dominantly exposed (102) facets. J. Phys. Chem. C.

[21] Xia J., Yin S., Li H., Xu H., Xu L., Xu Y. (2011). Improved visible light photocatalytic activity of sphere-like BiOBr hollow and porous structures synthesized via a reactable ionic liquid. Dalton Trans..

[22] Huo Y., Zhang J., Miao M., Jin Y. (2012). Solvothermal synthesis of flower-like BiOBr microspheres with highly visible-light photocatalytic performances. Appl. Catal. B Environ..

[23] Ye L., Liu J., Jiang Z., Peng T., Zan L. (2013). Facets coupling of BiOBr-g-C_3_N_4_ composite photocatalyst for enhanced visible-light-driven photocatalytic activity. Appl. Catal. B Environ..

[24] Cheng H., Huang B., Wang P., Wang Z., Lou Z., Wang J., Qin X., Zhang X., Dai Y. (2011). In situ ion exchange synthesis of the novel Ag/AgBr/BiOBr hybrid with highly efficient decontamination of pollutants. Chem. Commun..

[25] Shang M., Wang W., Zhang L. (2009). Preparation of BiOBr lamellar structure with high photocatalytic activity by CTAB as Br source and template. J. Hazard. Mater..

[26] Gao M., Zhang D., Pu X., Li H., Lv D., Zhang B., Shao X. (2015). Facile hydrothermal synthesis of Bi/BiOBr composites with enhanced visible-light photocatalytic activities for the degradation of rhodamine B. Sep. Purif. Technol..

[27] Yang C.-K., Naveenraj S., Lee G.-J., Wu J.J. (2015). Microwave-assisted synthesis of BiOBr microspheres for photocatalytic degradation of tartaric acids in aqueous solution. Top. Catal..

[28] Wang X., Yang J., Chen Y., Zhang Y., Tang Y. (2014). EDTA-modified hydrothermal synthesis of a novel four-leaf clover-shape BiOBr microstructure. Mater. Lett..

[29] Amin M., Anwar F., Janjua M.R.S.A., Iqbal M.A., Rashid U. (2012). Green synthesis of silver nanoparticles through reduction with *Solanum xanthocarpum* L. berry extract: Characterization, antimicrobial and urease inhibitory activities against *Helicobacter pylori*. Int. J. Mol. Sci..

[30] Loo Y.Y., Chieng B.W., Nishibuchi M., Radu S. (2012). Synthesis of silver nanoparticles by using tea leaf extract from *Camellia sinensis*. Int. J. Nanomed..

[31] Bindhani B.K., Panigrahi A.K. (2015). Biosynthesis and characterization of silver nanoparticles (SNPs) by using leaf extracts of Ocimum Sanctum L (Tulsi) and study of its antibacterial activities. J. Nanomed. Nanotechnol..

[32] Zhang Y., Yang D., Kong Y., Wang X., Pandoli O., Gao G. (2010). Synergetic antibacterial effects of silver nanoparticles@ aloe vera prepared via a green method. Nano Biomed. Eng..

[33] Elavazhagan T., Arunachalam K.D. (2011). Memecylon edule leaf extract mediated green synthesis of silver and gold nanoparticles. Int. J. Nanomed..

[34] Kumar K.M., Sinha M., Mandal B.K., Ghosh A.R., Kumar K.S., Reddy P.S. (2012). Green synthesis of silver nanoparticles using *Terminalia chebula* extract at room temperature and their antimicrobial studies. Spectrochimica Acta Pt. A Mol. Biomol. Spectrosc..

[35] Garg S., Chandra A., Mazumder A., Mazumder R. (2014). Green synthesis of silver nanoparticles using *Arnebia nobilis* root extract and wound healing potential of its hydrogel. Asian J. Pharma..

[36] Sangeetha G., Rajeshwari S., Venckatesh R. (2011). Green synthesis of zinc oxide nanoparticles by *aloe barbadensis miller* leaf extract: Structure and optical properties. Mater. Res. Bull..

[37] Prathna T., Mathew L., Chandrasekaran N., Raichur A.M., Mukherjee A. (2010). Biomimetic synthesis of nanoparticles: Science, technology & applicability. Biomimetics Learning from Nature.

[38] Ahmed S., Ahmad M., Swami B.L., Ikram S. (2016). Green synthesis of silver nanoparticles using *Azadirachta indica* aqueous leaf extract. J. Radiat. Res. Appl. Sci..

[39] Karthikeyan C., Ranjani M., Kim A.R., Yoo D.J. (2016). Synthesis of iron nanoparticles using *Azadirachta indica* extract and its catalytic activity toward nitrophenol reduction. J. Nanosci. Nanotechnol..

[40] Bhuyan T., Mishra K., Khanuja M., Prasad R., Varma A. (2015). Biosynthesis of zinc oxide nanoparticles from *Azadirachta indica* for antibacterial and photocatalytic applications. Mater. Sci. Semicond. Process..

[41] Krishnasamyet A., Sundaresan M., Velan P. (2015). Rapid phytosynthesis of nano-sized titanium using leaf extract of *Azadirachta indica*. Int. J. ChemTech Res..

[42] Mao X., Xie F., Li M. (2016). Facile hydrolysis synthesis of novel Bi_4_O_5_Br_2_ photocatalyst with enhanced visible light photocatalytic activity for the degradation of resorcinol. Mater. Lett..

[43] Zhang W., Sun Y., Dong F., Zhang W., Duan S., Zhang Q. (2014). Facile synthesis of organic–inorganic layered nanojunctions of g-C_3_N_4_/(BiO)_2_CO_3_ as efficient visible light photocatalyst. Dalton Trans..

[44] Zhang X., Wang C.-Y., Wang L.-W., Huang G.-X., Wang W.-K., Yu H.-Q. (2016). Fabrication of BiOBr_x_I_1−x_ photocatalysts with tunable visible light catalytic activity by modulating band structures. Sci. Rep..

[45] Stan M., Popa A., Toloman D., Dehelean A., Lung I., Katona G. (2015). Enhanced photocatalytic degradation properties of zinc oxide nanoparticles synthesized by using plant extracts. Mater. Sci. Semicond. Process..

[46] Wang Q., Niu T., Jiao D., Bai Y., Zhong J., Li J., She H., Huang H. (2017). Preparation of visible-light-driven BiOBr composites with heteropolyacids (H_3_PW_12_O_40_) encapsulated by a zeolite for the photo-degradation of methyl orange. New J. Chem..

[47] Di J., Xia J., Ge Y., Xu L., Xu H., Chen J., He M., Li H. (2014). Facile fabrication and enhanced visible light photocatalytic activity of few-layer MoS_2_ coupled BiOBr microspheres. Dalton Trans..

[48] Yuan D., Huang L., Li Y., Xu Y., Xu H., Huang S., Yan J., He M., Li H. (2016). Synthesis and photocatalytic activity of g-C_3_N_4_/BiOI/BiOBr ternary composites. RSC Adv..

[49] Du Q., Wang W., Wu Y., Zhao G., Ma F., Hao X. (2015). Novel carbon dots/BiOBr nanocomposites with enhanced UV and visible light driven photocatalytic activity. RSC Adv..

[50] Li W., Jia X., Li P., Zhang B., Zhang H., Geng W., Zhang Q. (2015). Hollow mesoporous SiO_2_–BiOBr nanophotocatalyst: Synthesis, characterization and application in photodegradation of organic dyes under visible-light irradiation. ACS Sustain. Chem. Eng..

[51] Waterhouse G.I., Bowmaker G.A., Metson J.B. (2001). The thermal decomposition of silver (I, III) oxide: A combined XRD, FT-IR and Raman spectroscopic study. Phys. Chem. Chem. Phys..

[52] Yu H., Liu R., Wang X., Wang P., Yu J. (2012). Enhanced visible-light photocatalytic activity of Bi_2_WO_6_ nanoparticles by Ag_2_O cocatalyst. Appl. Catal. B Environ..

[53] Di J., Xia J., Ge Y., Li H., Ji H., Xu H., Zhang Q., Li H., Li M. (2015). Novel visible-light-driven CQDs/Bi_2_WO_6_ hybrid materials with enhanced photocatalytic activity toward organic pollutants degradation and mechanism insight. Appl. Catal. B Environ..

[54] Lin H., Cao J., Luo B., Xu B., Chen S. (2012). Visible-light photocatalytic activity and mechanism of novel AgBr/BiOBr prepared by deposition-precipitation. Chin. Sci. Bull..

[55] Rathore J.S., Upadhyay M. (2013). Investigation of zinc concentration in some medicinal plant leaves. Res. J. Pharm. Sci..

[56] Bukhari H., Heba M., Khadijah Q. (2014). Ecofriendly dyeing textiles with Neem herb for multifunctional fabrics. Part 1: Extraction standardization. Int. J. Tech. Res. App..

[57] Liu Y., Zhu Y., Xu J., Bai X., Zong R., Zhu Y. (2013). Degradation and mineralization mechanism of phenol by BiPO_4_ photocatalysis assisted with H_2_O_2_. Appl. Catal. B Environ..

[58] Grabowska E., Reszczyńska J., Zaleska A. (2012). Mechanism of phenol photodegradation in the presence of pure and modified-TiO_2_: A review. Water Res..

[59] Ao Y., Wang P., Wang C., Hou J., Qian J. (2013). Preparation of graphene oxide–Ag_3_PO_4_ composite photocatalyst with high visible light photocatalytic activity. Appl. Surf. Sci..

[60] Fu H., Xu T., Zhu S., Zhu Y. (2008). Photocorrosion inhibition and enhancement of photocatalytic activity for ZnO via hybridization with C_60_. Environ. Sci. Technol..

[61] Ye L., Zan L., Tian L., Peng T., Zhang J. (2011). The {001} facets-dependent high photoactivity of BiOCl nanosheets. Chem. Commun..

[62] Cai L., Gong J., Liu J., Zhang H., Song W., Ji L. (2018). Facile preparation of Nano-Bi_2_MoO_6_/Diatomite composite for enhancing photocatalytic performance under visible light irradiation. Materials.

[63] Lin S.J., Ting J.M., Hsu K.C., Fu Y.S. (2018). A composite photocatalyst based on hydrothermally synthesized Cu_2_ZnSnS_4_ powders. Materials.

