# Supplementary material for: Facile Green Synthesis of BiOBr Nanostructures with Superior Visible-Light-Driven Photocatalytic Activity
Source: Materials (Basel). 2018 Jul 24;11(8):1273. doi: 10.3390/ma11081273 (PMC6117687; doi:10.3390/ma11081273)
Supplement: Supplementary file 1 [file materials-11-01273-s001.pdf]

Facile Green Synthesis of Biobr Nanostructures with Superior Visible-Light-Driven Photocatalytic Activity

Seema Garg*[1], Mohit Yadav[2], Amrish Chandra[3], Sameer Sapra[4], Soniya Gahlawat[4], Pravin P. Ingole[4], Milica Todea[5], Eniko Bardos[6], Zsolt Pap[5,7], Klara Hernadi[6]

Supplementary Materials

S1: In order to optimize the concentration of plant extract (P.E), BiOBr-G was synthesized with different amounts of plant extract i.e., 4 mL, 6 mL, 8 mL, 10mL, and 12 mL. The as-prepared samples were tested for the photodegradation of methyl orange (MO). From Figure S1, it can be clearly seen that the highest photocatalytic activity was observed with an 8 mL plant extract (P.E). Hence, 8 mL of plant extract was utilized for the experimentation.

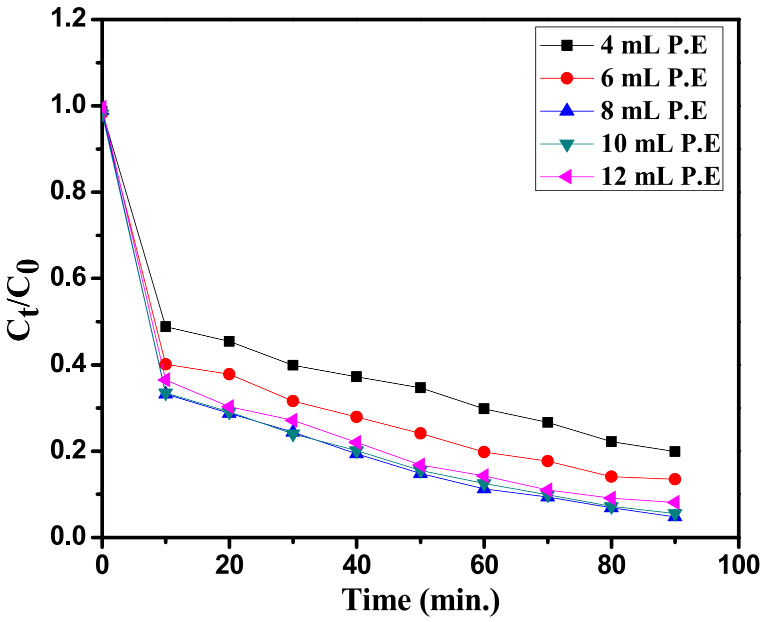

Figure S1. Photodegradation of MO by BiOBr-G with different concentrations of plant extract.

S2: Schematic representation of synthesis of green BiOBr using plant extract.

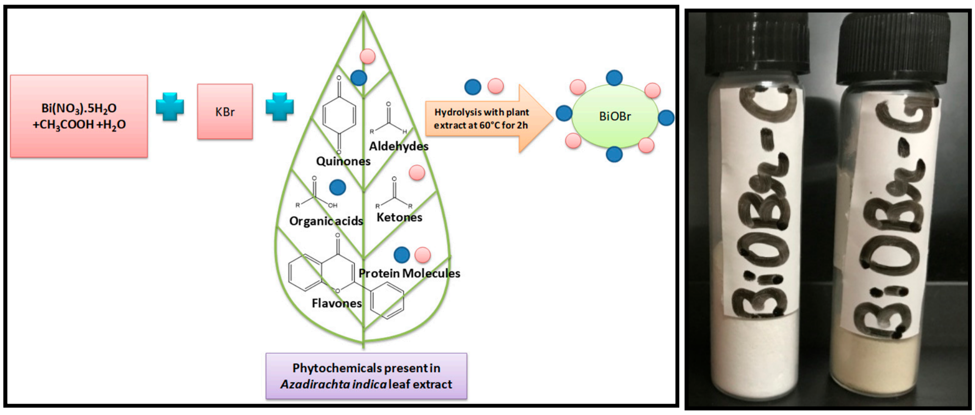

Figure S2. Schematic diagram for synthesis of BiOBr using *Azadirachtaindica leaf* extract and BiOBr-C and BiOBr-G samples.

S3: The dark experiment was conducted at different time intervals i.e., 30 min, 60 min, and 90 min. As shown in Figure S3, the highest adsorption occurred at 60 minutes and became constant at 90 minutes. Hence, adsorption–desorption equilibrium was attained in 60 minutes.

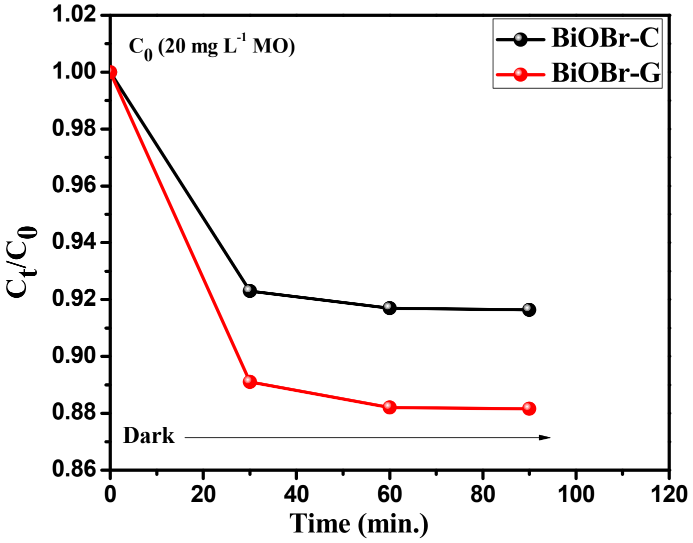

Figure S3. Showing the dark study at 30 min, 60 min, and 90 min for BiOBr-C and BiOBr-G.

S4: The average crystallite size of BiOBr-G and BiOBr-C was calculated using Debye Scherer's Equation.

$$D = K\lambda/(\beta \cos\theta) \quad (1)$$

where D is the size of the particles; K is the shape factor (0.9); λ is the wavelength of emitted x-rays (0.15418 nm); β is the full width at half maximum of the corresponding XRD peak; and θ is the angle of the incidence of the x-ray beam.

S5: This represents the corresponding TEM images of BiOBr-G and BiOBr-C (Figure S5). It can be clearly seen that the use of plant extract has introduced internal cavities in the mesoporous BiOBr-G nanostructures exhibiting a synergistic effect. Hence, the combined effect of internal cavities and lower

band gap of BiOBr-G facilitates the electronic transitions, subsequently resulting in higher photocatalytic activity under the visible light when compared to BiOBr-C.

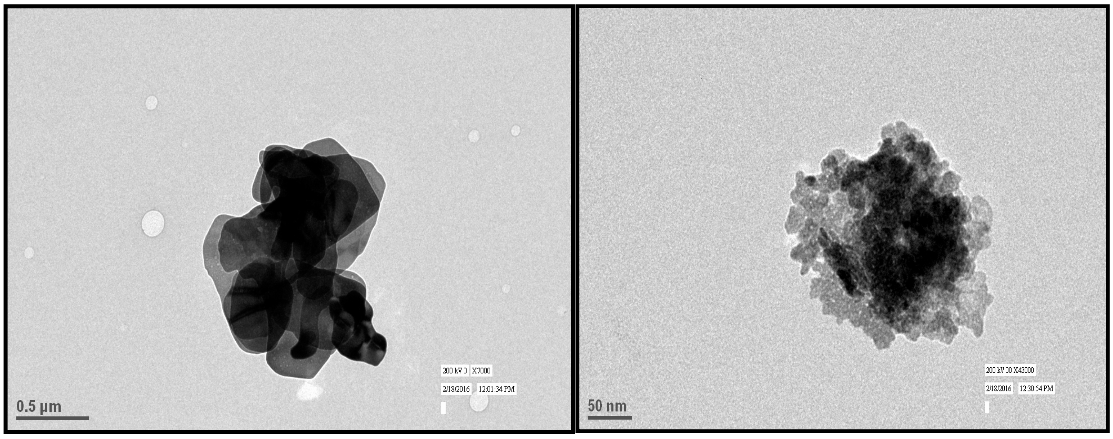

Figure S5. TEM images of (**left**) BiOBr-C and BiOBr-G (**right**).

S6: Photocatalytic Performance of Plant Extract

In order to investigate the role of leaf extract alone, a control experiment with raw leaf extract (10 mL) was conducted following the same method reported in the manuscript for the degradation of MO. As shown in Figure S6, the leaf extract did not influence any degradation of MO under 90 minutes of visible light irradiation. This suggests that the leaf extract alone does not possess any photocatalytic activity.

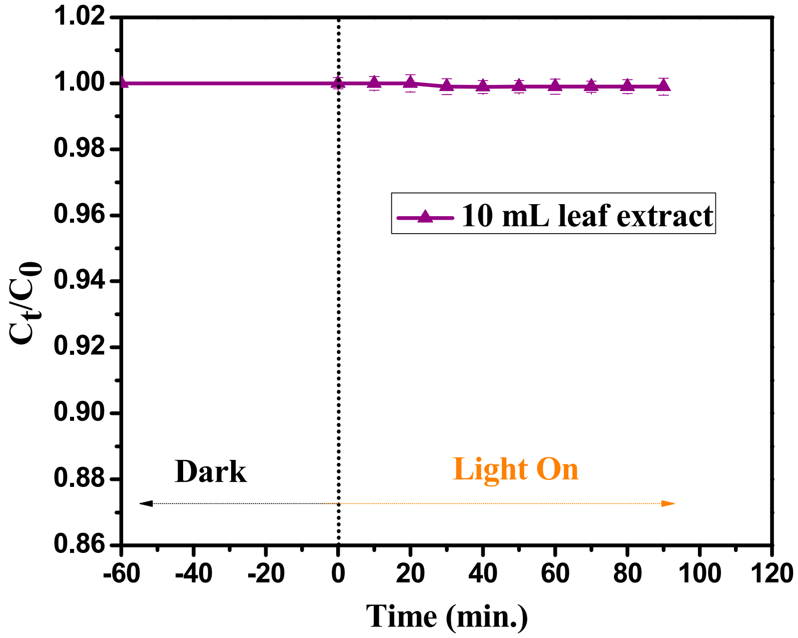

Figure S6. Degradation efficiency of MO with leaf extract.

S7: Photocatalytic Degradation of Phenol

For the prospect of dye sensitization, a typical colorless pollutant i.e., phenol was also chosen to further evaluate the photocatalytic activity of the as-prepared BiOBr-C and BiOBr-G. In the dark experiment, about 9.3 % and 13.6 % of the Ph was adsorbed on the BiOBr-C and BiOBr-G surface, respectively. Figure S6a shows that in the presence of BiOBr-C and BiOBr-G, the photodegradation rate of Ph was found to be 52.01 % and 68.67 % within 600 minutes, respectively. In addition, no change in the degradation efficiency of Ph was observed without the photocatalyst, indicating the stability of Ph under visible light irradiation. From Figures S6b,c it can be clearly seen that upon increasing BiOBr-C and BiOBr-G concentration from 50–150 mg, the photodegradation efficiency of Ph was further increased and was found to be 65.70% and 81.73%, respectively. In this study, 125 mg was found to be the optimized photocatalytic concentration in this case. Once again, the photocatalytic efficiency of BiOBr-G was found to be nearly 16 % more than that of BiOBr-C.

In order to investigate the active species responsible for Ph degradation, a similar study as that of MO was carried out in this case. As shown in Figures S6d,e, almost no inhibition of the photocatalytic performance was observed when isopropanol and benzoquinone were used to quench $\cdot OH$ and $\cdot O_2^-$. This indicates that $\cdot OH$ and $\cdot O_2^-$ showed a comparatively weak effect on the Ph degradation. However, a discerning inhibition of photocatalytic activity was seen when sodium oxalate was used to quench h^+ and confirms the importance of h^+ in the photooxidation process. Both BiOBr-G and BiOBr-C consumed more time in the degradation of Ph when compared to that of MO. This can be explained as in the case of MO, where all three active species actively participated in the photooxidation process while in the case of Ph; it wasonly the holes that were solely responsible for the degradation of Ph. Hence, using Equations (3),–(5), the photo-oxidation process that occurred in this case would be:

$$Ph + h^+ \rightarrow \text{degraded product} \tag{8}$$

Figure S6f shows the pseudo-first-order kinetic model for Ph degradation. The rate constants for BiOBr-C and BiOBr-G were calculated using Equation (7) as used earlier, and were found to be 0.0012 min^{-1} and 0.0019 min^{-1}, respectively. Interestingly, in this case, the calculated value of the rate constant for BiOBr-G was also greater than that of BiOBr-C, which explained the superior photocatalytic activity of BiOBr-G towards phenol.

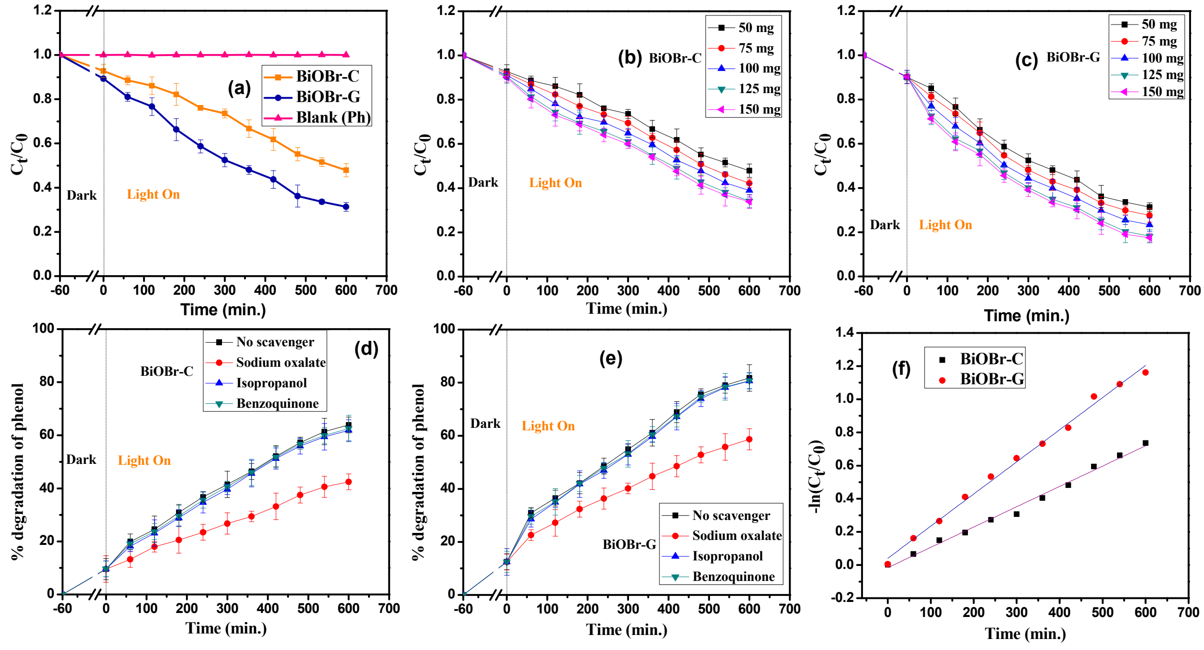

Figure S7. Photodegradation efficiency of Ph by BiOBr-C and BiOBr-G (**a**) with time, (**b**) with different concentrations of BiOBr-C, (**c**) with different concentrations of BiOBr-G. (**d**) Effects of different scavengers on degradation of Ph in presence of BiOBr-C (**e**). Effects of different scavengers on degradation of Ph in presence of BiOBr-G (**f**). Kinetic linear simulation curves of Ph over the samples.

S8: Band Position Calculations

For a compound, at the point of zero charge, the valence band (VB) position can be calculated by the following empirical formula[8]:

$$E_{VB} = X - E^{e} + 0.5\ E_{g} \qquad (9)$$

where X is the absolute electronegativity of the semiconductor, which is defined as the geometric mean of the absolute electronegativity of the constituent atoms; E^{e} is the energy of free electrons on the hydrogen scale (ca. 4.5 eV); E_{VB} is the VB edge potential ;and E_g is the band gap of the semiconductor. The conduction band (CB) position can be deduced by $E_{CB} = E_{VB} – E_g$. Given the equations above, the top of the VB and the bottom of the CB of BiOBr-G were calculated as 3.091 and 0.261 eV with respect to the normal hydrogen electrode (NHE), respectively. Accordingly, in the case of BiOBr-C, the VB and CB were estimated to be 3.196 and 0.156 eV, respectively.
